# Migraine Associated with Gastrointestinal Disorders: Review of the Literature and Clinical Implications

**DOI:** 10.3389/fneur.2014.00241

**Published:** 2014-11-21

**Authors:** Saskia van Hemert, Anne C. Breedveld, Jörgen M. P. Rovers, Jan P. W. Vermeiden, Ben J. M. Witteman, Marcel G. Smits, Nicole M. de Roos

**Affiliations:** ^1^Winclove Probiotics, Amsterdam, Netherlands; ^2^Division of Human Nutrition, Wageningen University, Wageningen, Netherlands; ^3^Department of Neurology, Gelderse Vallei Hospital, Ede, Netherlands; ^4^NijBarrahus Fertility Center, Wolvega, Netherlands; ^5^Department of Gastroenterology and Hepatology, Gelderse Vallei Hospital, Ede, Netherlands

**Keywords:** celiac disease, colic, gastroparesis, migraine, inflammatory bowel disease, irritable bowel syndrome, leaky gut, probiotics

## Abstract

Recent studies suggest that migraine may be associated with gastrointestinal (GI) disorders, including irritable bowel syndrome (IBS), inflammatory bowel syndrome, and celiac disease. Here, an overview of the associations between migraine and GI disorders is presented, as well as possible mechanistic links and clinical implications. People who regularly experience GI symptoms have a higher prevalence of headaches, with a stronger association with increasing headache frequency. Children with a mother with a history of migraine are more likely to have infantile colic. Children with migraine are more likely to have experienced infantile colic compared to controls. Several studies demonstrated significant associations between migraine and celiac disease, inflammatory bowel disease, and IBS. Possible underlying mechanisms of migraine and GI diseases could be increased gut permeability and inflammation. Therefore, it would be worthwhile to investigate these mechanisms further in migraine patients. These mechanisms also give a rationale to investigate the effects of the use of pre- and probiotics in migraine patients.

## Introduction

Migraine is a common headache disorder with a lifetime prevalence of 13% in men and 33% in women (1). There are ictal (migraine attack) and interictal periods. Migraine is a highly disabling disease with high personal and social costs (2). Migraine can be considered as a complex neurogenic inflammatory disorder (3–5) but the pathophysiology is still not fully understood (6). It is a disease of the brain, possibly of the brainstem and is associated with increased synthesis and release of calcitonin gene related peptide (CGRP). A migraine attack can be blocked with CGRP antagonists (3, 7, 8). The actual pain is generated by nociceptors of trigeminal nerve endings in the dura. Low serotonin levels may sensitize the nociceptors of trigeminal neurons (9). Existing data support that serotonin is low interictal but increased ictally in migraineurs (10, 11). Ictally serotonin agonist, like triptans and ergotamins, which decrease serotonin are associated with relief of acute pain (7–9). In contrast tricyclic antidepressants and selective serotonin and noradrenaline reuptake inhibitors, which are associated with increases in serotonin, are utilized for migraine prevention (11). Migraine attacks can be triggered by intrinsic cerebral factors (CGRP release), nitric oxide like tri-nitroglycerine, corticotrophin releasing hormone (stress), pro-inflammatory cytokines, and degranulation of mast cells located in the dura (1, 3, 8). Migraine has a genetic background, but the concordance in monozygotic twins is only 20%, indicating the importance of environmental factors in getting the disease (12).

An environmental factor that may play an important role is the gut microbiota. The number of bacteria in the human gut outnumbers the human cells by approximately 10:1 (13). Due to recent technical developments, studies of the gut microbiota are no longer dependent on culture techniques, but use high-throughput sequencing techniques to investigate the intestinal bacterial species. Over 25 different diseases are currently associated with alterations in the composition of the gut microbiota. At the moment, most attention has been given to inflammatory bowel diseases (IBDs), allergy, diabetes, and obesity (14). Besides gastrointestinal (GI) diseases, the gut microbiota as an independent factor can also contribute to systemic diseases. This can be caused by the migration of stimulated immune cells, by systemic diffusion of microbial products or metabolites, or by bacterial translocation as a result of decreased intestinal barrier function (15).

The brain and the GI tract are strongly connected via neural, endocrine, and immune pathways (16–18). The communication occurs in two directions, not only from the brain to the gut but also the other way around. This recent finding on the role of the gut microbiota in the gut-brain axis suggests that the gut microbiota can be associated with brain functions and neurological diseases like migraine. In this review, associations between migraine and GI diseases are studied and possible therapeutic consequences are hypothesized.

## Headache and Gastrointestinal Symptoms

Not all observational studies are restricted to migraine. The HEAD-hunt study, for example, looked at the relationship between GI symptoms and headache, including migraine (19). The study was a questionnaire-based cross-sectional study among more than 51,000 inhabitants of a county in Norway. The study showed a higher prevalence of headaches among people who regularly experience GI symptoms compared to the control group without GI complaints. The association between headache and GI complaints increased with increasing headache frequency. All the GI complaints were as common among persons with non-migrainous headache as among migraine patients. So both migraine and other types of headaches are more common in people with GI complaints.

## Migraine and Gastroparesis

Gastroparesis is a chronic disorder manifested by delayed emptying of the stomach. Gastroparesis is a relatively common complication of diabetes. In a population of patients with symptoms of diabetic gastroparesis, the patients with cyclic symptom patterns had a higher incidence of migraine headaches (47 vs. 20%, *p* = 0.02) compared to patients without cyclic vomiting pattern (20). Migraine attacks are associated with delayed gastric emptying (21). This migraine-associated gastroparesis is a problem for the treatment of the migraine with oral medicines, like oral triptans (22). Initially, delayed gastric emptying was found during migraine attacks, now there are also indications that in the interictal periods migraine patients have delayed gastric emptying. However, the studies done so far have been small and inconsistent in their results (23, 24), so further research in this topic is warranted.

## Migraine and Colic

Infantile colic is a common cause of inconsolable crying during the first months of life. It is defined according to criteria by Wessel as crying and fussing for more than 3 h per day, more than 3 days a week, and for more than 3 weeks in an otherwise healthy and well-fed infant (25). It affects many infants, with incidence rates ranging from 5 to 19% (26). Infantile colic might be caused by abdominal pain, although other causes cannot be excluded. A few studies have used probiotics to treat or prevent colic, with variable effects (27, 28). Colic has also been suggested as an early life expression of migraine, as in a group of 154 infant–mother pairs the children with a mother with a history of migraine (28 in total) were 2.6 times as likely to have colic as infants without maternal history of migraine (29). Recently, it has been shown that infants with abdominal colic have a lower intestinal microbiota diversity and stability compared to control infants in the first weeks of life (30). Moreover, children with migraine are more likely to have experienced infantile colic compared with controls (OR ranging from 1.6 to 6.6 between different studies) (31–33). Although long-term prospective studies have not yet been performed, these different studies indicate the existence of an association between migraine and infantile colic.

## Migraine and Irritable Bowel Syndrome

Irritable bowel syndrome (IBS) is a functional bowel disorder characterized by abdominal pain, bloating, discomfort, and marked changes in bowel habits as described in the ROME III criteria (34). The exact pathophysiology of IBS is not understood yet. IBS and migraine are both 2–3 times more prevalent in women than in men (1, 35–37). IBS has been shown to be a disorder with an increased intestinal permeability and this permeability increases with more severe IBS symptoms (38).

A study among approximately 125,000 IBS patients, identified in a large national health insurance database, found a prevalence of migraine of 60 per 1000 against 22 per 1000 in a control population from the same database (39). After correction for gender and age, and stratification of the mean monthly total medical cost, the odds for being diagnosed with migraine were 60% higher for people in the IBS cohort compared to people in the non-IBS cohort (39). Also other studies with smaller sample sizes indicated that 25–50% of IBS subjects had migraine, whereas it was only 4–19% in controls (40, 41). A meta-analysis showed that overall IBS patients are at risk to have coexisting headache with an estimated OR of 2.7 (CI 2.3–3.1) (42). Although no distinction between headache and migraine was made in this study, this suggests higher prevalence of migraine in IBS patients.

A study in Korean migraine patients revealed high numbers of functional GI symptoms in migraine patients, of which IBS related symptoms were the most common (43). Unfortunately, these numbers were not compared with control subjects, also in the total population a large percentage of the people fulfill the Rome III criteria for IBS.

Experimental evidence for an association between IBS and migraine comes from a study in which an IgG-based elimination diet was given to migraine patients with IBS. Twenty-one patients were included in the double blind, randomized, controlled, cross-over clinical trial with usual diet, elimination diet, and provocation diet (44). Compared with baseline, the elimination diet was associated with a significant reduction in migraine attack count, duration and severity. Also a significant reduction in IBS complaints was observed, demonstrating an association between the two diseases.

## Migraine and Celiac Disease

In patients who suffer from celiac disease, the immune system develops an autoimmune reaction against gliadin, the main protein in gluten. This inflammatory reaction is associated with intestinal damage, including dysfunction of the tight junctions resulting in an increased intestinal permeability (45–49). Celiac disease has been associated with migraine headache in case–control studies (50, 51). In one study, 14 out of 111 celiac disease patients (12.6%) reported migraine (50). The prevalence in controls was much lower, only 12 out of 211 controls (5.7%). In another study, migraine was present in 40 out of 188 celiac disease patients (21%), compared with 13 out of 178 controls (7%) (51). Conversely, there are also some indications that celiac disease is more frequent in migraine patients. One study showed that out of 90 adult migraine patients, 4 had celiac disease (4.4%), as opposed to only 1 out of the 236 controls (0.4%) (52). A second study in 72 pediatric patients with migraine showed four cases (5.5%) of elevated transglutaminase IgA antibodies. The transglutaminase IgA antibody level is a reliable indicator for the presence of celiac disease. Elevated transglutaminase IgA antibodies were found only in 1 out of the 147 controls (0.6%) (53). Another study among 87 pediatric migraine patients showed 1 child with celiac disease (1.1%), compared to 2 out of 543 controls (0.04%) (54). However, in another study no difference was found in the presence of celiac disease in 100 children with migraine and 1500 controls, being 2% for both groups (55). The association between migraine and celiac disease seems to be stronger in adult patients compared with children, although a direct comparison has not yet been investigated.

Only one study suggests that migraine in celiac disease patients may be relieved by treating celiac disease. Until now, the primary treatment for celiac disease is a gluten-free diet (56). The effect of a gluten-free diet was investigated in a small study with four patients with both migraine and celiac disease (52). In one patient migraine completely resolved. In the other three patients, a reduction in migraine frequency, duration, and intensity was reported. This suggests that a gluten-free diet used by celiac disease patients with migraine may give relief in both celiac disease and migraine. However, it should be noticed that only four patients were included in this study. Larger, well-designed studies to confirm these results are warranted.

## Migraine and Inflammatory Bowel Disease

The two main forms of IBD are ulcerative colitis and Crohn’s disease (57). These diseases are characterized by defects in the barrier function of the intestinal epithelial layer and the mucosal immune system (46, 57). Factors that may trigger IBD are antibiotics, non-steroidal anti-inflammatory drugs, stress, and infection. All these factors decrease the mucosal barrier integrity, modulate the immune response, and change the luminal microenvironment, providing the susceptibility to inflammation (58).

Data about possible correlations between migraine and IBD are scarce. To our knowledge, only two studies investigated the comorbidity between migraine and IBD. In the first study done by Ford et al., 100 patients with Crohn’s disease or ulcerative colitis were selected from the Gastroenterology clinic at the University of North Carolina (59). The prevalence of migraine in the IBD patients was 30%. This prevalence rate is higher than the US population basal rate of 18.2% for females and 6.5% for males. In the Crohn’s disease patients migraine was more prevalent (36%) than in the ulcerative colitis patients (14.8%) (59). In the second study, 111 patients with IBD were questioned in a survey (51). Prevalence of self-reported migraine was higher in these subjects compared with controls (OR 2.66, 95% CI 1.08–6.54). No reports in the literature were found showing a reduction in migraine frequency or severity with improvements of inflammatory bowel symptoms.

## Role for Gut Barrier Function in Migraine?

**Figure 1:**
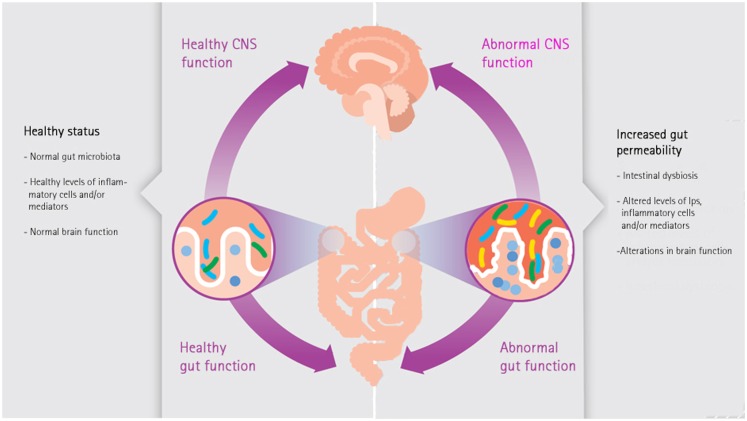
**The microbiota-gut-brain axis**.

This overview of the literature suggests the existence of a rather strong relationship between GI disorders and migraine. One of the links between inflammatory diseases and migraine are enhanced pro-inflammatory immune responses (60). In intestinal disorders characterized by an increased intestinal permeability like IBS, IBD, and celiac disease enhanced pro-inflammatory immune responses have been reported (48, 61, 62). Enhanced levels of pro-inflammatory cytokines like tumor necrosis factor alpha and interleukin 1β in serum of migraine patients have been found during migraine attacks (63). These cytokines can act on the nociceptors of the trigeminal nerve, causing migraine. Also statistical significant associations have been reported between migraine and a wide range of inflammatory disorders like asthma, obesity, metabolic syndrome, allergies, and GI diseases (4, 5, 64–70). A strong trigger of pro-inflammatory immune responses is the leakage of lipopolysaccharides (LPS) from the intestinal lumen into the circulation. Enhanced levels of LPS can enter the circulation when the intestinal permeability is increased (leaky gut, Figure 1). Depending on genetic susceptibility, pro-inflammatory responses can occur in different parts of the body, e.g., in case of migraine on the nociceptors of the trigeminal nerve.

Gut permeability and inflammation are bidirectional related, increased permeability can cause inflammation, but inflammation can also cause increased gut permeability (71). An increased gut permeability, and thereby increased translocation of LPS can be caused by multiple factors, like medicines, exercise, mast cell activation, high fat diet, stress, etc. (72). The most used method to measure epithelial barrier function is with the lactulose/mannitol test. Mannitol is transported via the transcellular pathway whereas lactulose is absorbed through the paracellular pathway. In case of increased permeability, more lactulose passes the barrier and eventually ends up in the urine. Therefore, an increase in intestinal permeability is characterized by an increased ratio of lactulose/mannitol (38, 73). It can be hypothesized that reduction of the permeability of the intestine results in relief of migraine in the subgroup of patients in whom intestinal permeability plays a role in the disease. One specific group might be migraine patients with food allergies. Subjects with food allergies have an increased intestinal permeability compared with healthy controls (74). The role of food allergens in migraine is controversial, as evidence linking avoiding suspected food triggers with improvement in migraine is still limited (60). In the 1990s and the first 10 years of this century, there has been almost no interest in studying the relationship between migraine and diet (75). However, some recent studies suggest a role for IgG-mediated food allergy in migraine (44, 76), a hypothesis that warrants further investigations.

## Treatment of Migraine with Probiotics?

Probiotics are living microorganisms that have beneficial effects on the health of the host (77). The most used probiotics are lactobacilli and bifidobacteria. Effects of probiotics are dependent on the used species and strain. Certain probiotics have shown to be effective in gut-related diseases, like infectious childhood diarrhea (78), the prevention of antibiotic-associated diarrhea (79, 80), and necrotizing enterocolitis in premature infants (81). For other clinical conditions, like atopic dermatitis, IBD, and IBS the results from clinical trials have been inconsistent (82, 83). This can be due to multiple factors like study population, duration, end-points, etc. An important variable are the different probiotic strains which are studied, making it difficult to draw conclusions about probiotics in general for these conditions. One of the possible working mechanisms of probiotics in the treatment of GI disorders is strengthening of the intestinal barrier. *In vitro* as well as *in vivo*, probiotics have shown to be able to improve the epithelial barrier function via different mechanisms (84, 85). Most mechanistic work has been done in cell culture systems or in animal models. In a randomized double-blind placebo-controlled cross-over study in healthy adults, probiotics were able to enhance the epithelial barrier by changing the location of the tight junctions proteins in the epithelial layer (86).

As probiotics may play a role in maintaining or improving gut barrier function in human beings, they can have a beneficial effect in migraine patients with an enhanced intestinal permeability as well. So far, no clinical randomized controlled trials have been published where migraine patients received nutritional therapy with probiotics. An uncontrolled study reported the effects of a combination of different probiotics (*Lactobacillus acidophilus*, *Lactobacillus bulgaricus*, *Enterococcus faecium*, and *Bifidobacterium bifidum*) with vitamins, minerals, micronutrients, and herbs in 40 migraine patients (87). At the onset of this study, the participants had a mean quality of life score of 38 [Medical Outcomes Trust Migraine Specific Quality of Life (MSQ) Questionnaire] and after 90 days of treatment their mean quality of life score was risen to 76. Sixty percent of the migraine patients experienced almost total relief from migraine attacks and they reported quality of life scores between 80 and 100.

## Conclusion

Next to migraine, other brain diseases have been suggested to be associated with increased gut permeability, including depression, autism, and stress (15, 88–90). There is growing interest in the role of the gut microbiota in these brain diseases. In this review, a possible route via an increased intestinal permeability is suggested. There is an accumulation of studies on both migraine and GI disorders (Table 1). However, the findings of some (small) studies are not supported yet by other independent studies. Nevertheless placebo-controlled studies in migraine patients using treatments directed at increased intestinal permeability are warranted. We have recently started a study in which permeability of the gut is measured in migraine patients as well as in controls. We also started a double blind, placebo-controlled trial to investigate the effect of a probiotic product on gut permeability as well as severity and incidence of migraine attacks. Hopefully, these studies provide an answer to the question if gut permeability plays a role in migraine patients.

**Table 1:** **Summary of intestinal diseases associated with migraine**.

Disease	Association
Gastroparesis	Present during migraine attacks, possible in interictal periods.
Colic	Children of migraine mother have more often colic; children with migraine have more often experienced infantile colic.
Irritable bowel syndrome	IBS patients have more often migraine; treatment of IBS might lower migraine.
Celiac disease	Celiac patients have higher prevalence of migraine; migraine patients might have more often celiac disease; migraine in celiac patients may be relieved by gluten-free diet.
Inflammatory bowel disease	IBD patients have a higher prevalence of migraine.

## Conflict of Interest Statement

Saskia van Hemert is employee of Winclove Probiotics. Winclove produces, markets, and investigates probiotics. Other authors declare that they have no conflict of interest.

## Abbreviations

CGRP, calcitonin gene related peptide; GI, gastrointestinal; IBD, inflammatory bowel disease; IBS, irritable bowel syndrome; LPS, lipopolysaccharides.
